# Supplementary material for: The sound of reading: Color-to-timbre substitution boosts reading performance via OVAL, a novel auditory orthography optimized for visual-to-auditory mapping
Source: PLoS One. 2020 Nov 25;15(11):e0242619. doi: 10.1371/journal.pone.0242619 (PMC7688106; doi:10.1371/journal.pone.0242619)
Supplement: Attachment — Submitted filename: Peer review.docx [file pone.0242619.s001.docx]

PLOS One – The Sound of Reading: Color-to-timbre substitution boosts reading performance via OVAL, a novel auditory orthography optimized for visual-to-auditory mapping.

Overall

This was an interesting and creative paper that explored the behavioral possibilities of sensory substitution in a new way, looking at how discrete sets of information can be conveyed rapidly and how its efficiency can be improved through specific design considerations centered around the auditory system. This is explored in the realm of learning to read via discriminating individual letters, and the writing largely reflects this topic. However, I believe that the implications of this work go beyond what is written and my suggested revisions primarily seek to further explore what is discussed and add new discussion points to reflect this broader focus.

Introduction

Para 2-3: The exploration of Braille literacy rates, achievement and its interaction with early/late-blindness is interesting and provides a good case for alternative means of information transmission. One wonders if the decline of Braille is also in part superseded by advances in text-to-speech software (JAWS, iPhone’s VoiceOver, Seeing AI), eliminating the effortful reading process from accessing the information (even if this comes with downsides). Is there any data that letter-by-letter reading literacy is still desired by the blind population when these alternatives are available?

“Initial attempts in this direction aimed at reading via embossed visual letters, which resulted in a very slow and cumbersome process.” I assume this is using Reference 27, which mentions that accuracy is reduced for embossed letters vs Braille, but nothing is noted about speed, so a reference backing up this point would be good.

Since you are teaching unique ‘letter-codes’ rather than written letter-shapes, there are going to be questions over how generalizable this approach is or what skills you are teaching subjects beyond a semi-arbitrary sound-letter memory task. For example, a Braille-EyeMusic conversion could not only teach subjects to auditory-read, but also teach Braille-dots for tactile-reading as well in terms of transferrable skills. While I understand OVAL is a preliminary exploration of auditory-reading designed around the auditory system, it would be good to make the potential transferrable impact of auditory-reading explicit for the reader (e.g. learning Braille, writing in OVAL, or familiarity with sensory substitution), whether or not this relates to OVAL specifically. This point could be made in the introduction or the discussion, or alternatively, that OVAL becomes an accessible language in of itself if justified. EDIT: In the method, its mentioned that OVAL is based off of Morse Code, hence you could say they are learning might be learning this as a transferrable skill as well.

“information.However” add space.

“Research in visual reading, demonstrated the opposite outcome, namely that when adding redundant discriminative features to each letter, such as color, reading performance was hindered (29).” Is this a study of the initial process of learning to read or with semi-proficient readers – only initial learning would be relevant to this study, so this caveat should be mentioned if it’s with with subjects with prior reading experience. EDIT: I see that you address this idea in the discussion “might facilitate letters discriminability in the initial stages of learning to read with a novel orthography.” But it’s still worth introducing this idea in the introduction as it seemed like a key point to make.

Participants

Please report the gender of the subjects for completeness.

Seems like a very small number of subjects (18), especially when this is further subdivided into two groups. While sample size is an easy critique to make, its particularly important to make it in this case. This should still be seen as a limitation and mentioned in the discussion (potentially within the context of future research studies).

OVAL system

“Letter orthography was based on the same combination of dots and lines as in Morse code… in OVAL lines were vertical rather than horizontal… This choice also allowed each character to be played more quickly than if horizontal lines were used” So here subjects only need to tell the difference between a single note (dot) vs multiple notes (dash) at a given moment (with the exception of the 4^th^ ‘dual’ component). I understand the rationale here but are vertical ‘dots’ and ‘lines’ in the EyeMusic easy for subjects to distinguish? Discriminating the presence/absence of multiple pitch-elements simultaneously from very brief exposure seems like it could be difficult, with Brown et al (2015; Pubmed ID: 26528202) showing that it is difficult for subjects to discriminate multiple pitch components when they are presented simultaneously and are harmonically-consonant (which all notes on the pentatonic scale are with each other). One way it is possible to examine these kinds of deficits is by looking for patterns of errors – e.g. are subjects more likely to mistake trained letters with similar numbers of components for one another in the monochromatic group (e.g. 4-component letters B, H, L, P, Q; 3-component letters D, U, W, S etc)? Do you have the data for what incorrect answers subjects gave for the identification of trained characters (Fig1.D)? A ‘confusion matrix’ would provide valuable insight into where subjects experienced difficulty. Furthermore, did the subjects report utilizing various strategies, or relying on specific sound qualities (especially in the monochromatic group), or complain about particular components being difficult?

Experimental procedure

“a letter discrimination task including both trained and untrained letters…” This was initially confusing as I was wondering how could subjects discriminate “untrained letters” when the shape and sonification of the letters is semi-arbitrary? From further reading, this appears to be a letter recognition task between trained and untrained letters. I believe this phrasing is clearer: a task discriminating trained from untrained letters.

Timbre not timber (this occurs twice in the manuscript)

“the 9 letters learned” There are different levels of repetition in learning for each of the letters – does this reflect their ability to do each letter? I understand there would also be a confound of auditory complexity since one of your rules is that more common letters involve more simplistic sonifications. This could also be learned from a ‘confusion matrix’ of what errors subjects made (especially in the monochromatic group).

While timbre/colour is used to differentiate different letters, it would be interesting to see if a ‘flash’ of colour could be used within a letter-sonification to further distinguish difficult letters (e.g. if a letter had 3 components, the middle one could be in a different timbre, or it could be used to help indicate ‘dashes’ rather than ‘dots,’ alleviating any potential difficulty in discriminating vertical lines. There are lots of options here worthy of future exploration, so this could be incorporated into the discussion.

Figure 1. Ortography or Orthography?

Exp1 (same/diff in pre vs post)

1-sentence paragraphs are not necessary.

“The 242 trials consisted of all possible combinations of the 11 experimental OVAL letters” This could be interpreted to mean that on 11% of trials the correct answer is ‘same’ and on 89% of trials the correct answer is ‘different’? However, in the results section it says chance was at 50%, so half the trials must have been ‘same’ and half ‘different,’ but there is not the information here for the reader to have known that. The make-up of the trails could be more explicit to the reader, so they would know this prior to the results section.

Exp2 (trained vs untrained – asked whether familiar or unfamiliar)

No comments

Exp3 (letter ID of trained characters)

No comments

Exp4 (reading novel words and pseudo-words – all trained letters)

How do you know they have correctly said the pseudo-word without spelling it out?

Data analysis

Single sentence paragraphs should be avoided again.

Results

**Exp1:**

Please report degrees of freedom within your t-tests (standard APA style).

Lowest p-value should be <.001, p-values should not be reported with an initial ‘0’ before the decimal point (because *p* cannot be above 1 it is redundant). ‘p’ and ‘t’ should be lowercase and italicized (e.g. *t*(8)=11.70, *p*<.001). Please apply these changes throughout.

“OVAL color readers’ average success was 99.7% (SD= 0.34%) (Figure 2A).” With such a high average score you probably hit the ceiling effect which means the test is becoming insensitive to the superior performance of the color group – while no methodological or reporting changes need be applied here, it would be a suitable discussion point as a shortcoming (alongside the small sample size).

“we performed a mix repeated measures ANOVA using average success rates as dependent variable…” minor grammatical changes to “we performed a mix**ed** repeated measures ANOVA using average success rates as **the** dependent variable…”

For ANOVAs, ‘partial eta squared’ should be reported as a measure of effect size for each of the main effects and interactions.

Otherwise it’s a good breakdown of the statistics and what they mean in the context of the study.

**Exp2:**

Cohen’s D starts being reported here, but it there are several versions of working this out – e.g. 1) The formula utilizes a specific groups’ SD as it is consider the control condition, or 2) a pooled-SD is averaged from both groups. It would be good to state which one it is, since the selection is not explicitly stated, I assume it’s the pooled-SD. This information should be added to the beginning of the results section.

**Exp3**:

“Indeed, while Monochromatic readers correctly identified the letters within about 2 repetitions of each character, Color readers required only one repetition on average” It would be good to get the mean average, rather than the mode.

**Exp4:**

Figures:

2A – ‘p’ in percent is cut off of the Y-axis

1C – Response speed shows a strange font

2F ‘1 repeatition’ misspelling

Discussion

Overall the discussion goes over some good points, but a lot of the time valuable space is used re-stating points the reader should already know (e.g. that the colour OVAL outperformed the monochrome OVAL) and that there are other, potentially more interesting points left unexplored.

As a reminder, the difficulty of discriminating multiple simultaneous notes (as discussed from Brown et al 2015 study) should be a discussion point since OVAL relies heavily on the dot/dash distinctions using vertical lines.

One potential future improvement (if there were more subjects) would be for a counter-balancing in which letters subjects are trained with. Although the rationale for selecting every 2^nd^ character works well for a single study.

**Auditory OVAL compared with tactile Braille**

‘The main mean’ – perhaps the main ‘method’ would flow better (there are multiple occurrences of this).

Morse code (as mentioned in the Introduction/method) might be a more suitable comparison for the effectiveness of OVAL than Braille, since they use the same dot/dash components. The other potential is that subjects might be able to transfer their OVAL learning to understanding Morse Code, which might be a good case study for how learning language in this manner could aspire to work in the future.

Designing for the auditory modality might also mean designing for the auditory expertise that the population by enlarge has from daily lived experience (not just musical notes that only musically-inclined have additional experience with) – for instance, vocal pitch, vocal sounds, distinctive auditory events.

“does not require to stretch the limits of human auditory perception” This is truer of the ‘colour’ OVAL than the monochrome one, but that might be because the redundant features are making up for mistakes the average listener has in terms of pitch discrimination – so in terms of pitch alone, I would say the monochrome OVAL might be at a difficulty slightly beyond the average novice’s pitch discrimination abilities (even if they could potentially train themselves to reach 100% scores with enough training).

“ultimately allowing participants to outperform the results obtained using the somatosensory system in the Braille discrimination tasks…” These are not always apples-to-apples comparisons here. Subjects were trained and assessed on 11 OVAL characters, it might be that Braille-training on 11 characters only might have resulted in similar outcomes.

“the more different characters are easily distinguishable among each other, the more learning is facilitated.” I noticed that all OVAL characters with multiple components *decreased* in pitch over time (with the exception of the 4^th^ component), is there something to be said for increasing variety/distinguishability through certain letters *increasing* or *maintaining* pitch over time, or is this a skill too specialized for the musically-inclined?

“understand the rules of spelling.” What would be the difference be between OVAL and a text-to-speech that converted individual letters rather than whole words, e.g. saying “h” “e” “l” “l” “o” rather than “hello.” Without the visuospatial conversion of OVAL, both would be arbitrary sound-letter associations, and both would help understand spelling. Is there any particular advantage to OVAL over this alternative (e.g. text-to-speech requires computer recognition)?

In terms of future devices / use cases, how well would OVAL work within a ‘live-viewing’ scenario – for instance, having the script written down on a page, and the subject using the iPhone EyeMusic to search for and read the script. Feeding a single image through the device is an ‘ideal’ scenario, but would the variations of sonification made by subjects ‘zooming’ into the letters (making them expand over more pixels) or having the grapheme lower/higher in the image (shifting the whole pitch-range down and up respectively) affect comprehension. Would subjects robustly learn the rules, or do subjects rely on exact pitches to solve these tasks?

**The boosting power of color information carried by sound**

“Will the facilitation be further enhanced by adding more colors/timbres?” I would see the ideal as trying various techniques to see how easily learnable a variety of monochromatic techniques are (e.g. trying rising pitches, or faster with more components etc), so that you have the best possible foundation before adding the timbre variations on top as well, which you already know enhance performance.

Building on when you mention that ‘color strengthens perceptual grouping’ of visually-presented words in sentences, colour could then be used to separate words in OVAL without the need for gaps of silence, potentially speeding performance.

“may stems” should be ‘may stem’

The serial vs parallel for auditory vs visual reading is a good point.

I would like to hear a discussion of some potential ‘end points’ for the OVAL line of research, what does an ideal system, device, or use case look like? Or, alternatively, is this purely a case study to prove a specific point on auditory reading? Could OVAL be a good replacement for Braille in everyday use scenarios?

It is surprising that what I believe is the key advantage of OVAL over many of the alternatives listed (Braille, Morse Code, text-to-speech; or my suggestion of letter-to-speech) is not emphasized, which is that of potentially transferrable skills since listeners are effectively learning a visuo-spatial to sound conversion algorithm and that OVAL might be an easy on-ramp to learning other skills with it (much like OVAL inadvertently teaches the subjects Morse Code), but that this overall skill can get subjects familiar with a versatile system that can be used for a multitude of additional purposes (e.g. learning Braille, ‘visually’-checking the writing of OVAL-characters, or writing in general).
