# Supplementary material for: The sound of reading: Color-to-timbre substitution boosts reading performance via OVAL, a novel auditory orthography optimized for visual-to-auditory mapping
Source: PLoS One. 2020 Nov 25;15(11):e0242619. doi: 10.1371/journal.pone.0242619 (PMC7688106; doi:10.1371/journal.pone.0242619)
Supplement: Attachment — Submitted filename: Peer_review_270920_final ra2.docx [file pone.0242619.s002.docx]

PLOS One – The Sound of Reading: Color-to-timbre substitution boosts reading performance via OVAL, a novel auditory orthography optimized for visual-to-auditory mapping.

October 2nd, 2020

## Re: Manuscript # PONE-D-20-15823

Title: The Sound of Reading: Color-to-timbre substitution boosts reading performance via OVAL, a novel auditory orthography optimized for visual-to-auditory mapping.

We very much appreciate the opportunity to revise the above manuscript and wish to thank Prof. Watanabe and the reviewers Giles Hamilton-Fletcher and Mike Richardson for the additional feedback which helped in improving the manuscript.

Below we list each point that was raised and indicate how we addressed it. The changes we made in the manuscript are noted in comments.

**Comment to the Editor:**

**The reviewers listed a number of specific points to be amended. In particular, the small sample size (N=18) is better to be justified (perhaps by performing a power-analysis) and the descriptions of statistics should be rechecked for the reviewers to properly evaluate a revised paper. Please provide point-by-point responses to the reviewers' comments.**

**Response:** We thank the editor for this important comments.

Sample size: A power analysis using G*power (Faul, Erdfelder, Lang, & Buchner, 2007, reference #57) showed that to achieve a power > 80% with a priori alpha set at 0.05 to study within-between variable interactions with partial eta squared of 0.14 in the ANOVAs, 5 participants are needed in each group. This information has also been added to the manuscript (p. 9).

Description of statistics, as also provided below as comments to individual queries by the referees, following the review we added the to the statistical analysis:

- Partial eta squared was added for all main effects and interactions in ANOVA tests.
- Description of Cohen’s d was added under the Data Analysis section.
- Results are now written in APA style.

**Regarding manuscript format:** we carefully formatted the revised manuscript according the PlosOne guidelines.

**Regarding Figure 1:** The copyrights for this figure are owned by the lab, as this figure was developed specifically for this manuscript by the lead author.

**Data availability:** Data can be found here: <https://osf.io/fu5tg/>

**Reviewer 1**

Overall

This was an interesting and creative paper that explored the behavioral possibilities of sensory substitution in a new way, looking at how discrete sets of information can be conveyed rapidly and how its efficiency can be improved through specific design considerations centered around the auditory system. This is explored in the realm of learning to read via discriminating individual letters, and the writing largely reflects this topic. However, I believe that the implications of this work go beyond what is written and my suggested revisions primarily seek to further explore what is discussed and add new discussion points to reflect this broader focus.

Introduction

**Para 2-3: The exploration of Braille literacy rates, achievement and its interaction with early/late-blindness is interesting and provides a good case for alternative means of information transmission. One wonders if the decline of Braille is also in part superseded by advances in text-to-speech software (JAWS, iPhone’s VoiceOver, Seeing AI), eliminating the effortful reading process from accessing the information (even if this comes with downsides). Is there any data that letter-by-letter reading literacy is still desired by the blind population when these alternatives are available?**

**Response:** We thank the reviewer for this comment. We now enlarged the discussion of the advantages of reading vs. VoiceOver converters and audiobooks in the introduction to the revised paper (see pp 4).

In brief, the seminal theory of the Simple view of reading, postulates that reading comprehension is determined by two cognitive capacities: decoding words through alphabetic coding, and language understanding (Gough & Tunmer, 1986; Hoover & Gough, 1990; see Kendeou et al., 2011 for more recent empirical evidence, references #35-37 in the revised manuscript). Under this framework, audiobooks (or VoiceOver) are not classified as reading as they lack the decoding aspect and only rely on language understanding. These predictions are corroborated by accumulating evidence documenting extensive changes triggered by literacy both in white and grey matter within the language-system, with reading activating a broader brain network compared to listening (Dehaene et al., 2015; Berl et al., 2010, references #1,41 in the revised manuscript). Nonetheless, evidence comparing audiobooks to print reading are still scarce, and more studies are needed to better uncover both differences and similarities between these two means of information acquisition.

Initial evidence also suggest that proper reading systems are preferred by blind readers over other assistive technology (AT) tools for information acquisition. Specifically, Argyropoulos et al. (reference #34 in the revised manuscript) investigated the preferred studying medium among 75 blind and visually impaired Greek individuals. It appears that proper reading systems, despite the more effortful process, were preferred by students, with 40% of the blind students choosing Braille as their preferred method for information acquisition, 59% of low vision students choosing large print, while screen readers, were chosen by only 1% of the participants. These results demonstrate that despite the high prevalence and availability of AT tools for information-acquisition, systems with active grapheme-phoneme correspondence are still preferred by students even if they slow down the speed of information acquisition, indirectly suggesting such systems provide an overall better experience also at the subjective level.

**“Initial attempts in this direction aimed at reading via embossed visual letters, which resulted in a very slow and cumbersome process.” I assume this is using Reference 27, which mentions that accuracy is reduced for embossed letters vs Braille, but nothing is noted about speed, so a reference backing up this point would be good.**

**Response:** We thank the reviewer for pointing out this inconsistency and indeed the reference provided measured only accuracy as an outcome without measuring recognition time. We corrected this in the revised manuscript (see p.5 in the revised manuscript). To the best of our knowledge, there is no evidence comparing accuracy and speed differences between embossed letters and Braille reading. We will be happy to add such a reference if the reviewer has one in mind.

**Since you are teaching unique ‘letter-codes’ rather than written letter-shapes, there are going to be questions over how generalizable this approach is or what skills you are teaching subjects beyond a semi-arbitrary sound-letter memory task. For example, a Braille-EyeMusic conversion could not only teach subjects to auditory-read, but also teach Braille-dots for tactile-reading as well in terms of transferrable skills. While I understand OVAL is a preliminary exploration of auditory-reading designed around the auditory system, it would be good to make the potential transferrable impact of auditory-reading explicit for the reader (e.g. learning Braille, writing in OVAL, or familiarity with sensory substitution), whether or not this relates to OVAL specifically. This point could be made in the introduction or the discussion, or alternatively, that OVAL becomes an accessible language in of itself if justified. EDIT: In the method, its mentioned that OVAL is based off of Morse Code, hence you could say they are learning might be learning this as a transferrable skill as well.**

**Response:** We thank the reviewer for raising the point of transferable skills, which helped in broadening the discussion.

Before diving into discussing transferable skills we would like to point that teaching Braille code via sensory substitution is indeed possible. Although never formally tested, and thus unpublished, we taught Braille via SSD in the lab prior to the development of OVAL, with the objective of comparing tactile vs. auditory recognition of the same characters (thus directly testing the role of the specific sensory modality on the speed of reading). The preliminary data we acquired suggest that the dot form of Braille is difficult to perceive via the auditory modality, at least when using the Eye-Music SSD, due to the low discriminability of separate dots on the same Y axis (which are translated into the auditory modality via SSD as two or three single-tones played at the same time; in line with results from the provided paper -Brown et al., 2015). Thus, we do not think that readers would benefit from auditory Braille (though perhaps it might be different with expert SSD users or perhaps with a different SSD algorithm). As far as Morse code goes – Morse code indeed uses more discriminable auditory features (long vs. short tones), but only uses one dimension of differentiation among character components (ie length of tone), and thus letters are of a longer duration which potentially limits reading speed. Nonetheless, given that OVAL letters use the same combination of lines and dots of Morse code (even though arranged differently), we indeed cannot exclude that learning OVAL might facilitate the learning of Morse.

We think that mostly, OVAL transferable skills mainly rely on sensory substitution learning. First, OVAL training may boost the tuning and sharpening of auditory skills, leading SSD users to successfully perceive tones of different frequencies played together (ie along the same Y axis; relates to the next comment as well), which are quite hard to perceive, and yet they are crucial for the perception of soundscapes of complex shapes. The OVAL system specifically addresses this specific skill with letters that contain a column with two components, most often a line and a dot (see Figure 1 of the revised manuscript where all OVAL characters are depicted). During training, special care was taken to teach to participants to perceive these two components, thus dissociating the two overlapping sounds. This skill could partially explain the improvement in the task of letter discrimination – before training some letters were perceived as identical while following training participants were able to identify the small differences among them. It should be noted that this improvement was most noticeable for the monochromatic group, as color letters were more discriminable even before any training. In addition, OVAL might be a novel, more engaging training approach for the initial stages of SSD learning.

We added a discussion on this topic in the revised manuscript (p. 23).

**“information.However” add space.**

**Response:** We thank the reviewer for this comment. We carefully proof-read the revised version of the manuscript and corrected mistakes were required.

**“Research in visual reading, demonstrated the opposite outcome, namely that when adding redundant discriminative features to each letter, such as color, reading performance was hindered (29).” Is this a study of the initial process of learning to read or with semi-proficient readers – only initial learning would be relevant to this study, so this caveat should be mentioned if it’s with with subjects with prior reading experience. EDIT: I see that you address this idea in the discussion “might facilitate letters discriminability in the initial stages of learning to read with a novel orthography.” But it’s still worth introducing this idea in the introduction as it seemed like a key point to make.**

**Response:** We thank the reviewer for this comment, indeed the study mentioned investigated proficient readers. We clarified this aspect in the introduction (see p.6 in the revised manuscript).

**Participants**

**Please report the gender of the subjects for completeness.**

**Response:** We added this information in the revised manuscript (see p.7).

**Seems like a very small number of subjects (18), especially when this is further subdivided into two groups. While sample size is an easy critique to make, its particularly important to make it in this case. This should still be seen as a limitation and mentioned in the discussion (potentially within the context of future research studies).**

**Response:** We thank the reviewer for this comment, and accordingly added in the discussion that future studies should include a bigger sample of participants.

**OVAL system**

**“Letter orthography was based on the same combination of dots and lines as in Morse code… in OVAL lines were vertical rather than horizontal… This choice also allowed each character to be played more quickly than if horizontal lines were used” So here subjects only need to tell the difference between a single note (dot) vs multiple notes (dash) at a given moment (with the exception of the 4^th^ ‘dual’ component). I understand the rationale here but are vertical ‘dots’ and ‘lines’ in the EyeMusic easy for subjects to distinguish? Discriminating the presence/absence of multiple pitch-elements simultaneously from very brief exposure seems like it could be difficult, with Brown et al (2015; Pubmed ID: 26528202) showing that it is difficult for subjects to discriminate multiple pitch components when they are presented simultaneously and are harmonically-consonant (which all notes on the pentatonic scale are with each other). One way it is possible to examine these kinds of deficits is by looking for patterns of errors – e.g. are subjects more likely to mistake trained letters with similar numbers of components for one another in the monochromatic group (e.g. 4-component letters B, H, L, P, Q; 3-component letters D, U, W, S etc)? Do you have the data for what incorrect answers subjects gave for the identification of trained characters (Fig1.D)? A ‘confusion matrix’ would provide valuable insight into where subjects experienced difficulty. Furthermore, did the subjects report utilizing various strategies, or relying on specific sound qualities (especially in the monochromatic group), or complain about particular components being difficult?**

**Response:** We thank the reviewer for this interesting and important comment as well as providing the reference which we now include in the manuscript (reference #66).

Based on the reviewer’s comment, we now included a “confusion matrix” in the revised manuscript for the identification letters task (Figure 2G). First, thanks to this comment, we noticed the letter B was marked as an experimental letter by mistake, while it was a control letter. We corrected this mistake in the revised version of the manuscript. The confusion matrix revealed no specific consistencies in the mistakes of the color group, while highlighted for the monochromatic group that the main confusion among monochromatic letters was between L and H (both 4 components letters), and U and W (both 3 components letters). Indeed these letters have somewhat similar spatial pattern (see Figure 1A in the revised manuscript). Interestingly this confusion was one-sided, so that participants mistook L for H and U for W but not the other way around. Overall, this confusion matrix excluded that mistakes of participants – even in the monochromatic group – were related mainly to distinguish between letters with 3 and 4 components, ultimately showing that OVAL training was successful in teaching participants to identify characters with 4 components. Specifically, during training participants were explicitly tutored to notice a dual component by targeting their attention to both lower and higher pitches. Second, and differently from the stimuli in the paper by Brown et al., OVAL stimuli compose a spatial pattern (rather than two discrete lines), and their identification requires the (implicit) identification of an entire pattern rather than specific attributes. Our results show that in most cases even when the spatial layout was similar participants were successful at telling the letters apart, ie successful at discriminating sounds on the Y axis. As correctly pointed out by this reviewer, differentiating components on the Y axis is indeed the more difficult auditory ability used in SSD. Thus, we now highlight more in the discussion of the revised manuscript, that OVAL can be a good tool for SSD training initiation, as it specifically teaches Y axis pitch discrimination (see p. 23 in the revised manuscript).

Regarding harmonically-consonant components – we again thank the reviewer for raising this issue. We suggest, that although all notes are harmonic in the sense they are on a pentatonic scale, the specific pattern of OVAL letters are not necessarily harmonic in the sense that the notes on the dual component do not comprise a legal accord. This could also explain how the notes were more discriminable than in the case of Brown et al.

As for specific complaints about letter being more difficult to discriminate: participants from the monochromatic group mainly complained about D and H (Dalet and Het), and U and W (Teth and Tzade). For the first pair, this was surprising since these two letters differ in their visual shape. Therefore during training we instructed participants to notice the dual component of H, and we taught them to hear the “falling down” nature of D (which starts with a line, thus gives the impression of a lower-tone pattern). As seen from the confusion matrix, by the end of training participants mastered this auditory discrimination skill. U and W confusion remained stronger even at the end of training. Future studies, with possibly longer training and bigger sample, can inform as whether this confusion improves with more training, and most importantly, if it affects word and text reading and comprehension.

**Experimental procedure**

**“a letter discrimination task including both trained and untrained letters…” This was initially confusing as I was wondering how could subjects discriminate “untrained letters” when the shape and sonification of the letters is semi-arbitrary? From further reading, this appears to be a letter recognition task between trained and untrained letters. I believe this phrasing is clearer: a task discriminating trained from untrained letters.**

**Response:** We thank the reviewer for this comment, and we changed the phrasing in the revised version of the manuscript.

**Timbre not timber (this occurs twice in the manuscript)**

**Response:** We corrected this in the revised version of the manuscript.

**“the 9 letters learned” There are different levels of repetition in learning for each of the letters – does this reflect their ability to do each letter? I understand there would also be a confound of auditory complexity since one of your rules is that more common letters involve more simplistic sonifications. This could also be learned from a ‘confusion matrix’ of what errors subjects made (especially in the monochromatic group).**

**Response:** We thank the reviewer for this comment. Indeed some letters were probably repeated more, both due to the step-wise nature of the training protocol, and because more common letters in the language are present in more words. However, we did not collect exact data on how many repetitions participants had with each letter. After the completion of the OVAL training protocol, participants freely read words from the entire pool and the trainer was encouraged to specifically choose words with specific letters participants had more identification issues with (while we could report some consistent difficulties with letters’ identification as discussed above, some variation between participants resulted in some participants initially confusing other letters). The confusion matrix that we created on the letter identification task shows that despite there might have been a small degree of variation in repetitions of OVAL characters over the entire training, end identification scores do not correlate with repetition during training. For instance, D and H were learned early in training but remained more difficult while Q, learned at the end of training, was easy to identify. Also, some letters (such as Q), despite having a relative difficult composition, were easy to identify due to their different overall pitch (Q is “higher” in general composition than other letters).

**While timbre/colour is used to differentiate different letters, it would be interesting to see if a ‘flash’ of colour could be used within a letter-sonification to further distinguish difficult letters (e.g. if a letter had 3 components, the middle one could be in a different timbre, or it could be used to help indicate ‘dashes’ rather than ‘dots,’ alleviating any potential difficulty in discriminating vertical lines. There are lots of options here worthy of future exploration, so this could be incorporated into the discussion.**

**Response:** We thank the reviewer for this comment. In the revised version of the manuscript we added the option suggested by this referee for manipulation of shape and color to further discriminate letters among various others (see p. 25 in the revised manuscript).

**Figure 1. Ortography or Orthography?**

**Response:** We changed into “Orthography”.

**Exp1 (same/diff in pre vs post)**

**1-sentence paragraphs are not necessary.**

**Response:** Corrected in the revised manuscript

**“The 242 trials consisted of all possible combinations of the 11 experimental OVAL letters” This could be interpreted to mean that on 11% of trials the correct answer is ‘same’ and on 89% of trials the correct answer is ‘different’? However, in the results section it says chance was at 50%, so half the trials must have been ‘same’ and half ‘different,’ but there is not the information here for the reader to have known that. The make-up of the trails could be more explicit to the reader, so they would know this prior to the results section.**

**Response:** We thank the reviewer for this comment. Indeed 11% of the trials were “same”, but since the participant had two options to answer (same\ different), the chance level was 50%.

**Exp2 (trained vs untrained – asked whether familiar or unfamiliar)**

**No comments**

**Exp3 (letter ID of trained characters)**

**No comments**

**Exp4 (reading novel words and pseudo-words – all trained letters)**

**How do you know they have correctly said the pseudo-word without spelling it out?**

**Response:** We thank the reviewer for this comment and by that stressing the difference between English and Hebrew. In Hebrew, spelling often corresponds to pronunciation. This is also true for pseudo-words. In the relatively few cases where the spelling was not certain from the pronunciation, we asked participants to spell the word in addition to the pronunciation. We made this point clear in the updated manuscript (see p. 13)

**Data analysis**

**Single sentence paragraphs should be avoided again.**

**Response:** We now avoided them in the revised manuscript

**Results**

**Exp1:**

**Please report degrees of freedom within your t-tests (standard APA style).**

**Response:** we now report degrees of freedom.

**Lowest p-value should be <.001, p-values should not be reported with an initial ‘0’ before the decimal point (because *p* cannot be above 1 it is redundant). ‘p’ and ‘t’ should be lowercase and italicized (e.g. *t*(8)=11.70, *p*<.001). Please apply these changes throughout.**

**Response:** We now report all the results according to standard APA style.

**“OVAL color readers’ average success was 99.7% (SD= 0.34%) (Figure 2A).” With such a high average score you probably hit the ceiling effect which means the test is becoming insensitive to the superior performance of the color group – while no methodological or reporting changes need be applied here, it would be a suitable discussion point as a shortcoming (alongside the small sample size).**

**Response:** We now added this consideration in the discussion. Although reaching the ceiling effect diminish tests’ sensitivity, this also suggests that if the two groups would be tested in harder tasks, the advantage of the color group over the monochromatic group might even be bigger (p. 24 in the revised manuscript).

**“we performed a mix repeated measures ANOVA using average success rates as dependent variable…” minor grammatical changes to “we performed a mix*ed* repeated measures ANOVA using average success rates as *the* dependent variable…”**

**Response:** We corrected the mistakes in the revised manuscript.

**For ANOVAs, ‘partial eta squared’ should be reported as a measure of effect size for each of the main effects and interactions.**

**Otherwise it’s a good breakdown of the statistics and what they mean in the context of the study.**

**Response:** We now report partial eta squared for all ANOVA results in the revised manuscript.

**Exp2:**

**Cohen’s D starts being reported here, but it there are several versions of working this out – e.g. 1) The formula utilizes a specific groups’ SD as it is consider the control condition, or 2) a pooled-SD is averaged from both groups. It would be good to state which one it is, since the selection is not explicitly stated, I assume it’s the pooled-SD. This information should be added to the beginning of the results section.**

**Response:** We thank the reviewer for pointing out the lack of specification regarding the calculation resulting in the reported Cohen’s d. Indeed, the reported number corresponds to the pooled-SD from both groups. Following this query, the information has now been added to the manuscript (pp.13-14).

**Exp3**:

**“Indeed, while Monochromatic readers correctly identified the letters within about 2 repetitions of each character, Color readers required only one repetition on average” It would be good to get the mean average, rather than the mode.**

**Response:** We thank the reviewer for this comment. We report the mean in seconds. As for the number of repetitions required for letter identification in each group, figure 2F in the revised manuscript directly presents this result by showing the percent of letters identified within 1 presentation, 1 repetition (ie. 1 presentation and another repetition), and >2 repetitions.

**Exp4:**

Figures:

2A – ‘p’ in percent is cut off of the Y-axis

1C – Response speed shows a strange font

2F ‘1 repeatition’ misspelling

**Response:** We thank the reviewer for this comment. We corrected the figures accordingly.

**Discussion**

**Overall the discussion goes over some good points, but a lot of the time valuable space is used re-stating points the reader should already know (e.g. that the colour OVAL outperformed the monochrome OVAL) and that there are other, potentially more interesting points left unexplored.**

**Response:** We now tried to limit repetitions in the discussion and added additional points to the discussion thanks to the interesting points raised by this reviewer and by reviewer 2.

**As a reminder, the difficulty of discriminating multiple simultaneous notes (as discussed from Brown et al 2015 study) should be a discussion point since OVAL relies heavily on the dot/dash distinctions using vertical lines.**

**Response:** We now added this point in the discussion in the revised paper (pp. 23-25).

**One potential future improvement (if there were more subjects) would be for a counter-balancing in which letters subjects are trained with. Although the rationale for selecting every 2^nd^ character works well for a single study.**

**Response:** We now added this suggestion to the discussion in the revised manuscript (p.19).

**Auditory OVAL compared with tactile Braille**

**‘The main mean’ – perhaps the main ‘method’ would flow better (there are multiple occurrences of this).**

**Response:** We changed it accordingly in the revised manuscript.

**Morse code (as mentioned in the Introduction/method) might be a more suitable comparison for the effectiveness of OVAL than Braille, since they use the same dot/dash components. The other potential is that subjects might be able to transfer their OVAL learning to understanding Morse Code, which might be a good case study for how learning language in this manner could aspire to work in the future.**

**Response:** We thank the reviewer for this comment, addressing the relationship between OVAL, Braille, and Morse code. OVAL indeed uses a combination of dots and lines similarly to Morse, with an important difference: the Morse code uses horizontal lines while OVAL uses vertical lines; consequently when OVAL letters are transformed to audition via SSD, they form a unique spatio-temporal pattern while Morse letters only vary in tone-length. Due to this spatiotemporal feature of letters, we think Braille reading is more appropriate for comparison. Indeed, although Braille letters are only composed of dots, letters do have a unique spatial pattern characterizing them. Another important reason for comparing OVAL with Braille is the prevalence of Braille as a reading tool (for blind, visually impaired and all others who cannot read visually), unlike Morse code that is used for reading only sporadically.

We added to the discussion of the revised manuscript that reading OVAL might facilitate future learning of Morse (although the two systems still differ in some aspects of the letters’ spatial arrangement) (see pp. 23-24 in the revised manuscript).

**Designing for the auditory modality might also mean designing for the auditory expertise that the population by enlarge has from daily lived experience (not just musical notes that only musically-inclined have additional experience with) – for instance, vocal pitch, vocal sounds, distinctive auditory events.**

**Response:** We thank the reviewer for this comment. In this context we would like to point that the white color of the EyeMusic is expressed via vocal pitch, even though EyeMusic users rarely recognize it as a “human” sound. It would be interesting to test in future studies customization of the EyeMusic for each user based on their sound preference.

**“does not require to stretch the limits of human auditory perception” This is truer of the ‘colour’ OVAL than the monochrome one, but that might be because the redundant features are making up for mistakes the average listener has in terms of pitch discrimination – so in terms of pitch alone, I would say the monochrome OVAL might be at a difficulty slightly beyond the average novice’s pitch discrimination abilities (even if they could potentially train themselves to reach 100% scores with enough training).**

**Response:** We thank the reviewer for this comment. We made this point clearer in the revised version. What we meant with this sentence was that Braille stretches the tactile acuity ability more than OVAL as it is clearly shown by the length of training programs aimed at teaching to read Braille. In the case of OVAL, even though participants, especially in the monochromatic group, significantly benefited from training, they were able to discriminate most of OVAL characters above the chance level also prior to specific OVAL training. This means that discrimination of OVAL characters is largely and relatively easily achieved by the human averagely trained auditory system.

**“ultimately allowing participants to outperform the results obtained using the somatosensory system in the Braille discrimination tasks…” These are not always apples-to-apples comparisons here. Subjects were trained and assessed on 11 OVAL characters, it might be that Braille-training on 11 characters only might have resulted in similar outcomes.**

**Response:** We thank the reviewer for this comment, and we agree that the comparison between our tasks and several other Braille studies is not always as straight-forward. In the present paper we cited several studies teaching novice Braille. These studies can be divided into those who taught only several letters of the alphabet in Braille for training duration that is similar to OVAL training (such as reference #51 by Millar et al), and to those who taught the entire alphabet but for a significantly longer duration of time (9 months, as in the case of reference #42, Bola et al.). In the study by Bola et al. for instance the first 3 months of training were dedicated only to letter discrimination, no reading or identification of letter took place during this time. Both these types of studies reported lower identification success rate in Braille characters’ identification compared to what we report for OVAL. Thus, these studies as a whole show that teaching Braille is a much slower process partially because of the difficulty with the tactile ability to perceive the nuanced differences between letters.

**“the more different characters are easily distinguishable among each other, the more learning is facilitated.” I noticed that all OVAL characters with multiple components *decreased* in pitch over time (with the exception of the 4^th^ component), is there something to be said for increasing variety/distinguishability through certain letters *increasing* or *maintaining* pitch over time, or is this a skill too specialized for the musically-inclined?**

**Response:** We thank the reviewer for this interesting comment. Indeed the OVAL characters are keeping with a pattern of decreasing pitch with the exception of the dual-tone component in letters with 4 components, with the idea of maintaining a unified pattern for all letters. While we haven’t formally tested how breaking this pattern of similarity will affect recognition, we included a “scrambled” version of OVAL characters as control in a follow-up experiment in fMRI on congenitally blind. In this experiment we tested the rapid neural recruitment for recognizing new script and used both unfamiliar (untrained) as well as scrambled characters as control. Although all participants commented how similar untrained letters were to the trained letters (ie all were decreasing in pitch), no neural difference was observed between untrained and scrambled characters. Mainly, all audemes that were not already associated with phonemes, were not perceived as letters by our participants. fMRI results fit with this behavioral result, as both unknown and scrambled letters showed no recruitment of ventro-occipital regions, unlike known letters, suggesting that the similarity per se of the OVAL characters’ sounds did not influence stimuli categorization. These results will be reported and discussed in a separate paper.

We nonetheless added in the discussion of the revised manuscript a paragraph stating that future studies should investigate the extent to which letters identification/reading performances would vary when the spatial patterns of OVAL characters would be modified (e.g., adding also “ascending” characters rather than using only “descending” characters as was the case here) (p. 25).

**“understand the rules of spelling.” What would be the difference be between OVAL and a text-to-speech that converted individual letters rather than whole words, e.g. saying “h” “e” “l” “l” “o” rather than “hello.” Without the visuospatial conversion of OVAL, both would be arbitrary sound-letter associations, and both would help understand spelling. Is there any particular advantage to OVAL over this alternative (e.g. text-to-speech requires computer recognition)?**

**Response:** We thank the reviewer for this interesting comment, raising the possible similarity between auditory patterns of OVAL characters and the auditory pattern of letters’ names (dictated spelling), as well as addressing the “audiospatial” nature of OVAL. Indeed it could be possible to spell each letter in a word separately, and thus essentially bind letter names to their phonemes as a method to convey information.

We think that dictated spelling will elicit a set of behaviors and neural activations that will lie in between the outcomes of audiobooks listening and the outcomes of reading. Dictated spelling, differently from audiobooks, requires both decoding words through alphabetic coding and language understanding, namely the two cognitive capacities determining reading comprehension according to the seminal theory of the Simple View of Reading (Gough & Tunmer, 1986; Hoover & Gough, 1990, Kendeou et al. 2009, references #35-37 in the revised manuscript). However, while in OVAL reading each character has a unique spatio-temporal patterns, which we believe facilitates perception and speeds comprehension, reading via dictated spelling relies strongly on visual imagery of the print alphabet of reference (which might not be similarly available to all users), in turn potentially making retrieving words more effortful. Note that dictated spelling might anyway prove to be a promising supportive training solution to teach the OVAL alphabet, or other non-visual orthographies, mediating the passage from the print visual alphabet to non-visual orthographies. We added this paragraph in the discussion of the revised manuscript (see pp. 22-23).

**In terms of future devices / use cases, how well would OVAL work within a ‘live-viewing’ scenario – for instance, having the script written down on a page, and the subject using the iPhone EyeMusic to search for and read the script. Feeding a single image through the device is an ‘ideal’ scenario, but would the variations of sonification made by subjects ‘zooming’ into the letters (making them expand over more pixels) or having the grapheme lower/higher in the image (shifting the whole pitch-range down and up respectively) affect comprehension. Would subjects robustly learn the rules, or do subjects rely on exact pitches to solve these tasks?**

**Response:** We thank the reviewer for this interesting comment, which we will respond to in two levels – first by addressing the behavioral/ technical aspects, and second the cognitive aspect.

The reviewer raises the question about using OVAL with everyday devices. We already developed a first OVAL app for Android devices which works that when a user wants to read a paragraph, the app would automatically extract the text and translate it directly and automatically into OVAL, not as a picture, but as a text-to-OVAL converter. This has already been implemented as an SMS reading app, in beta, for our participants from the lab. The app also uses the gyroscope in the phone to change speed of reading (users can rotate the phone up or down to increase or decrease reading speed). So to address the technical aspect of the question, when translating a digital written text into OVAL, the letters themselves do not need to change. The difference between OVAL and JAWS (for example) remains to be the active reading vs. passive listening with its potential neural and comprehension advantages. Our users also like the privacy they get with OVAL vs. listening to text messages (while in public, this requires to wear headphones all the time to avoid people eavesdropping).

The second part of the question relates to the potential invariability of the audio-spatial pattern to changes in pitch/speed. Visual print reading has been shown to be invariant to changes in font, size and location in the visual field (Zhou et al. 2019, reference #60 in the revised manuscript), which has been suggested as one of the characteristics of automatic/ holistic reading. We indeed conducted a follow-up experiment with expert EyeMusic users who practiced OVAL reading. From the results obtained we can say that similarly to print, participants internalized the whole pattern of each letter, independent from specific pitch, and could identify letters when location, size and speed were manipulated. This results will be reported in a follow-up paper that also addresses the neural recruitment following OVAL reading acquisition.

**The boosting power of color information carried by sound**

**“Will the facilitation be further enhanced by adding more colors/timbres?” I would see the ideal as trying various techniques to see how easily learnable a variety of monochromatic techniques are (e.g. trying rising pitches, or faster with more components etc), so that you have the best possible foundation before adding the timbre variations on top as well, which you already know enhance performance.**

**Response:** We agree with the reviewer that improvement in OVAL discriminability should also address the arrangement of components instead of only adding more timbres. We discussed this aspect in the revised manuscript. We think that there is not necessarily a hierarchy of more/less relevant features to modify in the OVAL alphabet. We now suggest that future studies should inform us on which combinations of color and/or shape variations enhance characters discriminability the most (p. 25).

**Building on when you mention that ‘color strengthens perceptual grouping’ of visually-presented words in sentences, colour could then be used to separate words in OVAL without the need for gaps of silence, potentially speeding performance.**

**Response:** We agree with the reviewer that color could be used to separate words. Indeed, following up on this experiment, with a group of congenitally blind, we continued to train them to read whole sentences with OVAL, interesting topics from Wikipedia based on their interest etc (not always as part of a methodological study, as participants liked OVAL and the possibilities it opens for them, even though most were fluent in Braille). With the program we developed in the lab, spaces between words and sentences could be manipulated to increase and decrease speed. In general and from the experience we gathered, space between words was not the main issue in fluent reading, but the need to sometimes go back and re-read a word/ sentence. In visual reading, readers often use saccades to go back to previously read words, and the same is true for tactile reading in Braille, with readers going to the previous word with their finger, or when reading with two hands – the second hand re-reads the words. Thus from our experience thus far, building fluency mostly required the possibility to easily go back and re-read a word/ sentence. This option has been built into the app we developed specifically for OVAL.

**“may stems” should be ‘may stem’**

**Response:** Corrected

**The serial vs parallel for auditory vs visual reading is a good point.**

**I would like to hear a discussion of some potential ‘end points’ for the OVAL line of research, what does an ideal system, device, or use case look like? Or, alternatively, is this purely a case study to prove a specific point on auditory reading? Could OVAL be a good replacement for Braille in everyday use scenarios?**

**Response:** We thank the reviewer for this important comment.

We developed OVAL with two goals in mind. The first is for rehabilitation and providing reading alternatives, mainly for the blind and visually impaired population, but also for other populations such as people with reading disabilities. We would like to stress the we believe literacy should be made accessible to everyone, in their preferred method. In line with this, we believe Braille should be learned and made accessible to all individuals who wish to use it and can benefit from it. The problem is, less than 10% of blind actually read and use Braille in everyday life, which points toward an emerging issue of illiteracy in blindness. We believe OVAL can fill part of this gap by providing means for literacy that are relatively easy and cheap to master. As mentioned above, our end point is to make OVAL an option for digital devices as it requires no separate hardware that can be cumbersome and expensive. In our mind’s eye, OVAL can even be used with Kindle and other digital readers, to provide blind and visually impaired the option to read any book or text they want. In this way OVAL can be read in parallel with print (in case of visually impaired) as a dual-sensory approach to reading.

The second aim of OVAL, is to provide an easy and straight-forward approach for the cognitive study of non-visual reading. While the neural underpinning of reading and reading acquisition has been studied extensively in sighted, for what concerns non-visual reading in-depth investigations are lacking. One of the stumbling blocks for this line of research is the difficulty in mastering Braille and the technical difficulty in haptic devices in fMRI scanners. We believe OVAL can be used to study non-visual reading and the development of the multi-modal nature of the reading system. Indeed, we conducted such a study with congenitally blind adults. The results obtained from this separate study will be reported in a follow-up paper.

We now added more clearly these aforementioned points in the conclusions of the revised manuscript (pp. 28-29).

**It is surprising that what I believe is the key advantage of OVAL over many of the alternatives listed (Braille, Morse Code, text-to-speech; or my suggestion of letter-to-speech) is not emphasized, which is that of potentially transferrable skills since listeners are effectively learning a visuo-spatial to sound conversion algorithm and that OVAL might be an easy on-ramp to learning other skills with it (much like OVAL inadvertently teaches the subjects Morse Code), but that this overall skill can get subjects familiar with a versatile system that can be used for a multitude of additional purposes (e.g. learning Braille, ‘visually’-checking the writing of OVAL-characters, or writing in general).**

**Response:** We thank the reviewer for this important comment and agree with it, and consequently we added a discussion about transferable skills (p. 23 in the revised manuscript).

**Reviewer #2:**

*The manuscript introduces OVAL, a character-based visual-to-audio sensory substitution algorithm based on the existing eyeMusic application. OVAL is a character system, or auditory character based reading system, that uses the eyeMusic for processing the ‘audemes’. The authors posit this as a tool to help non-literacy in non-visual readers, in response to the dwindling numbers of Braille readers. The authors test the efficiency of OVAL with 18 sighted participants after training for 6 hours. The participants were split into two groups, monochromatic and colour, to see whether a greater range of timbres could help further improve auditory reading. The results suggest that colour information that is subsituted into timbre can help add 'texture' and improve reading accuracy and speed. The paper suggests that OVAL can be learnt faster than Braille and may assist in the learning of non-visual reading.

I liked the paper a lot and think it makes a valuable contribution to the field. I particularly like the demonstration of colour-to-timbre substitution for improving the speed and accuracy of performance, as colour substitution is generally under-represented (or even written off!) in SSD research. More work into using colour as an extra layer of distinguishing multiple features apart will be really useful for the field.*

**I do think the narrative needs to be tweaked slightly. At the moment, there feels like there is a large focus is on the practical uses of OVAL, in that, it has the potential to be more useful/available/easier to learn than Braille, and the authors make a compelling case for as such. However, with such a focus on the practical aspects of OVAL, there needs to be an earlier and thorough address of how OVAL stands apart from screen readers and audiobooks. It is only late in the discussion that screen readers or audiobooks gets mentioned, but I spent the whole paper thinking about them.**

**Response:** We thank the reviewer for this suggestion. We now added a paragraph discussing the difference between typical phoneme-to-grapheme reading systems and screen readers or audiobooks (see p. 4 of the revised manuscript).

**The authors mention how Braille is dying out, but I would possibly say that this could be related to the increased availability of audio technology. I know that reading through OVAL is a different skill, and will offer different cognitive development to listening to spoken word, but this should be made very clear. I would like to see some specific comparisons of past literature about the general benefits of reading (for knowledge, information etc.) against the benefits of specifically reading through characters (spelling, grammar etc.). Basically, I want to know in more detail why the declining Braille literacy is a problem, and why screen readers and audiobooks can’t fully solve this problem. Is Braille dying out simply because audiobooks and screen readers are easier and quicker to attain? This would do well in the introduction, so that your readers are immediately on board with why OVAL is important, and why blind and visually impaired readers should use it at the cost of slower text acquisition.**

**Response**: We thank the reviewer for this comment. We now added a dedicated paragraph on this important topic in the introduction to the paper (see p. 2;4 of the revised manuscript):

**Also, the authors need to say why audemes are better than just playing audio recordings of spoken individual letters. Each letter has its own sound and would represent an ‘audeme’ that is already familiar. Is OVAL just like learning a purely auditory character language?**

**Response**: We thank the reviewer for this comment, that also relates to the response provided above. The main purpose of literacy is to establish a functional link between phonological and orthographic representations which in turn exerts a cascade of effects which benefit both brain organization and behavior. According to the Simple View of Reading theory (referenced #35,36 in the revised manuscript), reading comprehension results from two processes: 1 decoding of letters through alphabetic coding and 2. language comprehension. Following this definition, listening to single letters might result in typical reading outcomes. Our hypothesis, nonetheless, is that a dedicated orthography such as OVAL might be more effective than playing each individuals letter’s name (dictated spelling) because each character of the OVAL has a unique spatio-temporal patterns which we believe facilitates perception, while reading via dictated spelling strongly relies on visual imagery of the alphabet of reference (which might not be similarly pronounced in all users) and thus retrieving words may result in being more effortful. Our hypothesis is that this latter reading solution will elicit a set of behaviors and neural activations that will lie in between the outcomes of audiobooks listening and the outcomes of reading. However, to the best of our knowledge, such hypothesis was never directly tested so future studies should address it directly and test the extent to which listening via method of playing each letter’s name separately might be a promising venue for further developing non-visual reading (perhaps also as a supportive training method to teach the OVAL alphabet or other auditory orthographies). We now added this explanation also in the discussion of the revised manuscript (pp. 22-23).

**I suspect the PLOS ONE will address formatting issues through publication, but if you need to use the accepted version for anything, I would recommend checking titling and heading formats in the paper as there are some inconsistencies. For a specific example, in the methods section, behavioural experiments (page 16), ‘Experiment 1 – letter discrimination’ has no capitalisation on the ‘l’ but ‘Experment 2 – Discrimination of trained vs. untrained letters’ has capitalisation on the ‘d’. Also, there’s the typo on ‘experiment 2’. Then later on in the manuscript, in the results section (page 18), ‘Experiment 1 - Letter discrimination task’ is not in italics which doesn’t pair with the methods section to well, and the ‘l’ is now capitalised.

I would also check formatting with reporting statistics in text. For example, on page 19, the manuscript has a mixture of spacing between statistic, operator, and number: ‘([F(1,16) = 11.3, p = 0.004])’ and ‘=11.70, p<0.00001; Color readers t=158.55, p<0.00001’. I would also check general conventions for reporting statistics. On PLOS ONE’s guideline page they have some useful info. Like reporting p-values less than 0.001 as p < 0.001 rather that trailing zeros, and stating whether one or two tailed test were used. It’s really handy for readers to be reminded of effect sizes too. On page 20, I assume you’re using cohen’s d, remind the audience what is considered a big/medium effect size. Also, double check which statistics should be in italics. Here’s the guide as a starting point:** [**https://journals.plos.org/plosone/s/submission-guidelines.#loc-statistical-reporting**](https://journals.plos.org/plosone/s/submission-guidelines.#loc-statistical-reporting)

**Response**: we thank the reviewer for pointing out formatting issues, which we now addressed in the revised version of the manuscript.

**Those are the main points that need addressing, and here are some specifics:

I think having the first line of the introduction being the same as the first line of the abstract damages readability a little bit, consider rewording?**

**Response**: we thank the reviewer for pointing this out. We now rephrased the first line of the introduction.

**On page 11, in the participants section, I think it’s useful to briefly justify why sighted participants rather than blind. Other researchers in the area will understand why, but those reading from outside may question why a Braille-like system is being tested with sighted.**

**Response**: We thank the reviewer for pointing this out. We added this information in the revised manuscript (see p. 6)

“…For this first investigation into auditory reading we tested sighted rather than blind participants to assess whether our auditory orthography is a valid non-visual reading system. This allowed us to exclude possible confounds in our results such as the level of literacy (all our participants acquired reading at typical developmental stages) or auditory training and experience (which is known to be enhanced in the blind population (53–56)). These results can be used as a baseline for quick OVAL learning, which could be then used in future studies as comparison to results acquired in other populations such as blind and visually impaired, as well as synesthetes or people with reading difficulties, among others…”

**Page 14, 2 hours for each training session seems like a long time, maybe add a note on participants fatigue or if there was a break.**

**Response:** We thank the reviewer for this comment, and added this information on page 9 of the revised manuscript:

**”** During each two-hour sessions, participants took short breaks when they felt tired to prevent excessive fatigue and to allow the maintenance of focus during OVAL learning”

**Page 17, I don’t think you need to repeat ‘i.e., the time used by the EyeMusic to scan each OVAL display’. After the first time, it was clear that four second per trial was due to the speed of the scan.**

**Response:** we agree with the reviewer and omitted redundant repetitions of this sentence in the revised version of the manuscript.

**Page 18, state which JASP version you used if available.**

**Response:** JASP version is now added (version 0.11.1)

**Page 19, ‘This suggests that even without any phonological association to the auditory patterns, basic auditory discrimination of the soundscapes was greatly enhanced by the addition of color conveyed via timbre manipulations(see also (32)for another color-related advantage in soundscapes discriminations)’ – I would get rid of this from the results and move the discussion. This sounds like interpretation of the results, rather than raw results.**

**Response:** We agree with the reviewer and avoided interpretation of results within the results section in the revised version of the manuscript.

**Page 20, there’s a disparity between the reaction time and character repetition. The manuscript states that both groups required one character repetition, but their response times were both under 4 second. Do you mean one character presentation rather than repetition? The character was presented one time only rather than repeated. This would make sense with the reaction times, otherwise I would expect the reaction times to be over 4 seconds.**

**Response:** Indeed, we meant only one presentation was needed. We corrected this in the revised manuscript.

**Page 22, ‘Finally, and most importantly, also the interaction between…’. Again, I feel like the word 'importantly' is adding some interpretation to the raw results. There hasn’t been any justification for this being more important than the other results.**

**Response:** We thank the reviewer for pointing this out, and in the revised manuscript we avoided such interpretation of the results within the results section.

**Discussion – I think the authors need to tone down some of the claims slightly. Namely, that the results demonstrate that audio reading can be ‘mastered’ in adulthood. It’s too earlier to say whether your participants mastered OVAL reading; a longer, more in depth training procedure would be needed to see possible performance. Actually, this claim does a disservice to the study, as with a lot more practice your participants may get much better! The discussion also goes into detail about accuracy in Braille reading studies, but not speed of reading. Previously, the manuscript mentions that the colour OVAL does not suffer with a speed accuracy trade off (so speed of reading is important), but then neglects speed comparisons with Braille.**

**Response**: We thank the reviewer for this comment. Consequently, we downplayed the conclusion in the revised manuscript and we now say that the current results *suggest* that audio reading can be mastered in adulthood and that future studies should test further this intriguing conclusion with additional OVAL training.

Regarding speed of reading: due to the overall difficulty in mastering Braille reading, studies on Braille reading acquisition require significantly longer training period before participants achieve functional reading abilities and thus tests on reading speed can only be conducted after significantly longer training duration. For instance, in study by Bola et al., highly motivated participants (such as Braille teachers) learned to read tactile Braille for 9 months, with the first 3 months dedicated to Braille character discrimination. This already shows that OVAL is much faster to learn, and thus direct comparison with speed of Braille reading is significantly biased based on the different scale of training duration.

Second, when speed of reading is discussed in Braille reading in the context of significantly more prolonged training (on the scale of months and years), authors typically report how many letters can be decoded by readers in a minute. In the current study that aimed at investigating rapid reading acquisition on the scale of hours, we did not collect this measure. Specifically each letter was presented within an interval of 4 seconds due to the scanning time of the EyeMusic which in this first study on the topic was fixed. Consequently the measurement of words-per-minute cannot be extrapolated. Taken together, in the present case we cannot conclude about the speed of reading in the way it is generally discussed in Braille studies and therefore we do not directly address this topic in our discussion, but only in the broader qualitative view of how the speed of learning to read differs between the two reading methods in light of the vastly different lengths of training.

Finally, when discussing the lack of speed-accuracy trade-off, we only compared speed of reading between our experimental groups. The color group was both more accurate and quicker than the monochromatic group, thus excluding the possibility that the addition of color to OVAL creates a speed-accuracy tradeoff in readers’ performances.

We added in the discussion of the revised manuscript that future studies should address reading speed with OVAL (see p.19).

**The declining Braille section (page 25) should potentially be in the introduction. While the intro does mention that Braille is declining it doesn’t give these specifics which would help justify OVAL slightly more thoroughly. Also, later on the page, the sentence ‘Several previous studies conducted on blindfolded sighted Braille learners, provide convergent results supporting these conclusions’ needs references at the end of the sentence.**

**Response**: As suggested by this reviewer in one of the comments above, we added a more thorough explanation in the introduction to the paper on the reasons behind the need of creating alternative-to-Braille orthographies (see p 4 in the revised manuscript).

As for the mention of the lack of references, the following paragraph with references # 58, 65, 59, goes in detail about how studies on sighted support this conclusion. Based on the referee’s comment we changed the dot into a colon.

**Page 27, I really like this section. It would be cool to see any comparisons to synesthetes reading if there’s any applicable research! Do colour-grapheme synesthetes have increased comprehension because of colour information, like with OVAL? Not sure if this has been done to be honest.**

**Response**: We agree with the reviewer that this is an interesting question. To the best of our knowledge even though several studies showed various advantages related to color-grapheme synesthesia, no one investigated the effect of this type of synesthesia on reading comprehension. We added this suggestion to the discussion (see p.27 of the revised manuscript):

“One interesting study to address this issue might be to test color-grapheme synesthets and ask whether they would also show better reading performances similarly to OVAL readers, at least during reading acquisition.”

**Page 30, the authors need to address reading comprehension here. Slow reading (even in dyslexics) reduces comprehension, and the relative slowness of reading by OVAL (compared to slighted reading) may actually hinder reading comprehension*.***

**Response**: We agree with the reviewer that slowing too much the reading process might hinder rather than facilitate reading comprehension. However please note that the OVAL speed can be adjusted and made quicker. In the case of this study we set each scanning of an EyeMusic image to 4 seconds. Advanced versions of the system used for leisure reading already have a dedicated app in which the presentation time can be individually adjusted to match reading speed of each individual. This includes both the speed of presentation of each letter (that can be even doubled) as well as the adjustment of spacing between letters and words. Additionally to keep with the flow of both Braille and print reading, the app allows users to repeat words and sentences thus providing a faster and smoother reading experience. In relation to dyslexic readers, dyslexic children are reported to read in one year the amount of words that a typical reader reads in 2 days (Cunningham and Stanovich 1988, reference #86 in the revised manuscript). So all researchers agree that the main challenge is to bring dyslexics to read more words in less time. Evidence suggests for instance that increasing spacing between letters improve reading skills in dyslexics because it reduces crowding, a perceptual phenomenon known to be defective in dyslexics (Zorzi et al. 2012, reference #82 in the revised manuscript). We suggest that adding auditory letters may help in facilitating letter recognition ultimately contributing to speed-up the extremely slow reading time characterizing dyslexics.

We added these considerations more clearly in the revised manuscript (see pp. 28-29).

**Figures are great! Just check capitalisation again, some variables start with capital letters and other don’t.**

**Response**: We now checked and corrected the font style of all figures.
